# Cross-sectional evaluation of a peer educator-led empowerment initiative for improving participation in cervical precancer screening among female sex workers in Ghana

**DOI:** 10.3332/ecancer.2025.1869

**Published:** 2025-03-11

**Authors:** Kofi Effah, Ethel Tekpor, Comfort Mawusi Wormenor, Joseph Emmanuel Amuah, Vida Kwawukume, Louisa Ademki Matey, Seyram Kemawor, Stephen Danyo, Esu Aku Catherine Morkli, Nana Owusu Mensah Essel, Emmanuel Timmy Donkoh, Patrick Kafui Akakpo

**Affiliations:** 1 Catholic Hospital, Battor, PO Box 2, Battor, via Sogakope, Volta Region, North Tongu, Ghana; 2 School of Epidemiology and Public Health, Faculty of Medicine, University of Ottawa, 451 Smyth Road (2046), Ottawa, ON K1H 8M5, Canada; 3 Pro-Link, DTD KA 5028, Spintex Highway, East Airport, Accra, Ghana; 4 Ayawaso West Municipal Health Directorate, PO Box YK 1484, GA 160-8888, George Walker Bush Highway, Kanda, Accra, Ghana; 5 Department of Emergency Medicine, College of Health Sciences, Faculty of Medicine and Dentistry, University of Alberta, 730 University Terrace, Edmonton, AB T6G 2T4, Canada; 6 Screen and Treat Research Group, Center for Research in Applied Biology, School of Sciences, University of Energy and Natural Resources, PO Box 214, Sunyani, Ghana; 7 Department of Pathology, University of Cape Coast, School of Medical Sciences, Clinical Teaching Center, Private Mail Bag, GN-06035819, Cape Coast, Ghana

**Keywords:** cervical precancer, female sex workers, screening, peer education, health service utilization

## Abstract

**Purpose:**

Despite having an increased risk of HPV infection, female commercial sex workers (FCSWs) have low uptake of cervical screening. Among FCSWs in the Volta Region of Ghana referred for screening following peer education, we examined the prevalence of high-risk human papillomavirus (hr-HPV) and cervical lesions and modelled factors associated with these screening outcomes.

**Patients and methods:**

As part of the mPharma Ten Thousand Women Initiative, implemented between September and October 2022, 340 FCSWs in Ho and Aflao were recruited by peer educators. Screening involved MA-6000 testing (with full genotyping using the AmpFire HPV platform) and enhanced visual assessment (EVA) mobile colposcopy.

**Results:**

The FCSWs screened had a mean age of 31.9 (SD, 9.3) years. The prevalence of cervical lesions on mobile colposcopy was 9.8% (95% confidence intervals (CI), 4.9–14.7), while the prevalence of hr-HPV infection among the FCSWs was estimated at 28.0% (95% CI, 20.6–35.3). The commonest individual genotype was HPV68 (9.1%; 95% CI, 4.9–15.0), while the least common genotypes were HPV33 and HPV39 (each 0.7%; 95% CI, 0.0–3.8). FCSWs screened in Aflao maintained threefold higher odds of hr-HPV infection (aOR, 3.33; 95% CI, 1.18–9.40) and having clinically significant EVA findings was associated with approximately fivefold higher odds of hr-HPV infection (aOR, 4.64; 95% CI, 1.09–19.83; *p*-value = 0.038). Condom use at the time of screening was independently associated with decreased odds of hr-HPV infection (aOR, 0.14; 95% CI, 0.04–0.49; *p*-value = 0.002). HIV infection (aOR, 9.95; 95% CI, 1.19–83.37) and engagement in oral-genital contact (aOR, 5.56; 95% CI, 1.35–22.94) were associated with higher odds of clinically significant EVA findings.

**Conclusion:**

Our study underscores the urgent need for comprehensive and targeted interventions to reduce the burden of hr-HPV and the potential for cervical precancer/cancer among FCSWs in Ghana. We further highlight the importance of promoting continuous condom use among FCSWs during interactions with patrons and considering HIV status in the development and implementation of cervical precancer screening programs for FCSWs.

## Introduction

Cervical cancer ranks fourth in prevalence among women worldwide, accounting for an estimated 660,000 new cases and 350,000 fatalities in 2022 [1]. Although a decrease in cervical cancer incidence has been reported in many high-resource countries, its prevalence and incidence continue to increase among women in a majority of low- and middle-income countries. Nowhere is this more apparent than in the sub-Saharan region. In Ghana specifically, it is the second most common cancer among women, contributing to 19.2% of all newly diagnosed cancer cases and 10.1% of cancer-related deaths in 2022 [2]. In both high- and low-resource settings, occupational exposure to multiple sexual partners places female commercial sex workers (FCSWs) at a higher risk of sexually transmitted infections (STIs), including high-risk human papillomavirus (hr-HPV) infection, and thus a higher risk of cervical precancer and cancer [3, 4]than women in the general population. Transmitting hr-HPV types to their male patrons also increases their susceptibility to penile cancer [5, 6]and their likely interaction with foreign patrons and sex tourists may play a key role in altering the prevailing local HPV genotype diversity in a country.

Although the exact number of FCSWs in Ghana is currently unknown, it is thought to be increasing in urban centres and was estimated at approximately 60,000 in 2020 [7, 8]. In the broadest sense, FCSWs in Ghana are a heterogeneous and itinerant population with limited access to routine healthcare services. Street-based FCSWs (‘roamers’) solicit clients on streets, in alleys, and parks, while the majority (‘seaters’) are more reclusive, often employed in brothels, massage parlours and lodges [12, 13]. The activities of FCSWs are criminalised in Ghana [9–11]. As a result, FCSWs are vulnerable to unlawful treatment from patrons in the form of violence, rape, ‘condom sabotage’ (intentional damage of condoms by clients), homicide and harassment/blackmail by law enforcement agents [13, 14]. In addition, they remain exposed to the risk of STIs (such as HIV and HPV infection) as a result of younger age at sex debut, engagement in risky sexual behaviours such as unprotected sex and illicit drug use [8, 10–13, 15, 16].

Cervical precancer screening in Ghana is not yet covered under the National Health Insurance Scheme (NHIS); thus, most women, and by extension FCSWs, are less likely to seek screening since they have to pay out of pocket. Again, most FCSWs are of low socioeconomic status and likely to seek care at advanced stages of the disease when any treatment (e.g., radical surgery or radiotherapy) would be suboptimal and expensive [3], leading to a poorer prognosis. Thus, efforts at expanding screening to this vulnerable group and treating precancerous lesions where necessary are paramount to lowering the associated morbidity and mortality.

In Africa, the need to tailor hr-HPV and cervical cancer prevention efforts to FCSWs is increasingly being recognised. Furthermore, continuous evaluation of the prevalence, associated factors and prevailing landscape is imperative. For instance, a review of facility and community-based reproductive health interventions directed toward FCSWs revealed that a majority of services were small-scale, poorly coordinated, had scanty government investment and focused more on HIV prevention and ancillary testing for STIs and less on HPV [17]. Broader reproductive testing, condom use and utilization of reproductive health services among FCSWs have been largely overlooked [18–25]. Pro-Link is one of a few non-governmental organizations (NGOs) that have emerged to bridge the gap by providing equitable reproductive health services to vulnerable groups in Ghana, including women living with HIV (WLHIV) and FCSWs, often using networks of peer educators [26]. While the role of peer educators has been harnessed in implementing cervical precancer screening programs among FCSWs in other sub-Saharan countries like Cameroon [3], to the best of our knowledge, no prior study has described the use of peer educators in carrying out cervical precancer screening and treatment in this vulnerable group in Ghana.

Under the Ghana arm of the mPharma 10,000 Women Initiative (mTTWI) [27], the Cervical Cancer Prevention and Training Centre (CCPTC), Catholic Hospital, Battor, Ghana partnered with Pro-Link to offer cervical cancer prevention services to two cohorts: WLHIV and FCSWs. Following the successful implementation of the mTTWI, the lessons learned and findings for a separate cohort of WLHIV have been published elsewhere [27]. In this paper, we primarily aimed to examine the prevalence of hr-HPV infection and cervical lesions among FCSWs screened under the mTTWI using concurrent hr-HPV DNA testing and mobile colposcopy and to assess factors associated with these screening outcomes. Secondarily, we describe the lessons learned and field reflections that enabled us to successfully carry out cervical precancer screening in this vulnerable and high-risk group using peer educators as a bridge between FCSWs and practitioners.

## Materials and methods

### Study design and setting

Among FCSWs screened between September and October 2022, with funding from the Ghana arm of the mTTWI and through peer education activities carried out by Pro-Link, we conducted this descriptive retrospective cross-sectional study to evaluate the prevalence of hr-HPV infection and cervical lesions. We further modelled factors associated with hr-HPV infection and cervical lesions as screening outcomes. The study participants were one of several special groups screened under the mTTWI, a collaborative project spearheaded in Ghana by the CCPTC, with the larger aim to provide 10,000 women in Ghana and Nigeria (who would ordinarily be unable to afford to do so) with complimentary cervical precancer screening by way of hr-HPV DNA testing (using the MA-6000 platform) [28, 29]. In both settings, cervical precancer screening was performed using concurrent testing, defined as a combination of hr-HPV DNA testing and visual inspection (with enhanced visual assessment (EVA) mobile colposcopy) in a single visit.

The FCSWs were screened in Aflao and Ho, both in the Volta Region of Ghana. Aflao is located in the southeastern corner of the Ketu-South Municipality and is often described as the eastern gateway to Ghana because it borders Lomé, the capital of Togo. It has a vibrant nightlife, two major markets and a good road network and thus attracts business from all over Ghana, Togo, Benin and Nigeria. Ho is the administrative and commercial capital of the Volta Region. It has a large market that attracts business from all over the region and people from neighbouring Togo. The town is home to three hospitals, including one teaching hospital. The town is accessed by well-paved roads and recently had an airport completed and opened to commercial traffic in 2021.

### Peer educator engagement and FCSW recruitment

The intervention was successfully implemented via collaboration with Pro-Link, an NGO dedicated to promoting and protecting the general and reproductive health of FCSWs in many cities in Ghana. The main activities of Pro-Link involve providing HIV testing and helping HIV-positive FCSWs’ navigate care and treatment (either at a drop-in centre or during mobile outreaches) while scheduling HIV-negative FCSWs for retesting quarterly.

Generally, the four peer educators recruited for the intervention were self-identified FCSWs who had received education on cervical cancer prevention and screening and so could share this information with their contemporaries in a culturally sensitive and nonjudgmental manner. They had 4–8 years of peer-educating experience. The peer educators were also available to motivate and accompany their peers to access screening services at designated clinics, where they received counselling, testing and treatment if necessary. After screening, they also provided follow-up support and aided in referrals for further care if needed.

Before visiting the study sites, peer educators used brochures and posters to facilitate health education and peer mobilization. The peer educators then handed each FCSW a referral card carrying her name and the name of the referring peer educator. During the onsite visits for screening, nurses from the CCPTC reiterated this education on STI prevention, HPV and cervical cancer. The major criteria for inclusion considered asymptomatic women aged ≥25 years who self-identified as FCSWs. Due to the expected high risk of hr-HPV genotypes in the study cohort, we also allowed some FCSWs who were younger than this cutoff age to participate if they were parous. We excluded FCSWs who were pregnant, who had undergone prior total abdominal hysterectomy and those who had already been diagnosed with invasive cervical cancer.

### Ethical considerations

This study was conducted in accordance with the Declaration of Helsinki (1964) and its later amendments. Verbal informed consent was sought from the women before questionnaire administration, collection of cervical samples and additional screening procedures. The consent procedure was approved by the Ethical Review Committee of the Catholic Hospital, Battor (approval no. CHB-ERC 0120/06/22), which also permitted the researchers to publish the study findings retrospectively.

### Sample size

This empowerment initiative was originally conducted as part of service provision to the FCSWs and not in a research context. Therefore, the study involved a convenience sample, including all FCSWs in Ho and Aflao referred by peer educators through Pro-Link Ghana for screening by nurses from the CCPTC under the mTTWI between September and October 2022.

### Data collection and handling

After providing verbal informed consent, participant information was captured using a structured electronic questionnaire in REDCap version 11.0.3 (Vanderbilt University, Nashville, TN, USA) by nurses from the CCPTC in a private room. The collected data were then securely stored within databases hosted and managed by the CCPTC. Prior to any analyses, the database was queried and the information was extracted and converted into an Excel spreadsheet (Microsoft Corporation, Redmond, WA, USA). Data accuracy was ensured by manual cross-checking. The anonymity and privacy of the participants were upheld by data deidentification.

### Study variables and outcomes

We collected sociodemographic data such as age, educational status, marital status, parity, occupation, level of income, residence, NHIS coverage, religion and parity. Data regarding self-reported risk factors were also collected, including details on current and past contraceptive use, smoking status (ever or current) and HIV status. With regard to their day-to-day activities as FCSWs, data collected included how long one had worked as an FCSW (only for those screened at Aflao), number of clients/patrons per week (only for those screened at Aflao), use of vaginal herbal preparations and type(s) of sexual activity engaged in. The screening outcomes of interest were a positive hr-HPV DNA test and the presence of clinically significant cervical lesions observed on visual inspection procedures (EVA mobile colposcopy).

### Screening procedures: cervical specimen collection, EVA mobile colposcopy and hr-HPV DNA testing

During the screening session, each FCSW was counselled on the benefits of cervical precancer screening as well as the potential risks and outcomes involved. In the dorsal lithotomy position, a sterile speculum was passed to adequately expose the cervix for sampling of the transformation zone (TZ) using a cytobrush or cotton-tipped applicator. The collected specimen was then placed in a labelled collection tube, sealed, stored at room temperature and submitted for testing within 1 week at the central laboratory of the Catholic Hospital, Battor.

**Figure 1.**
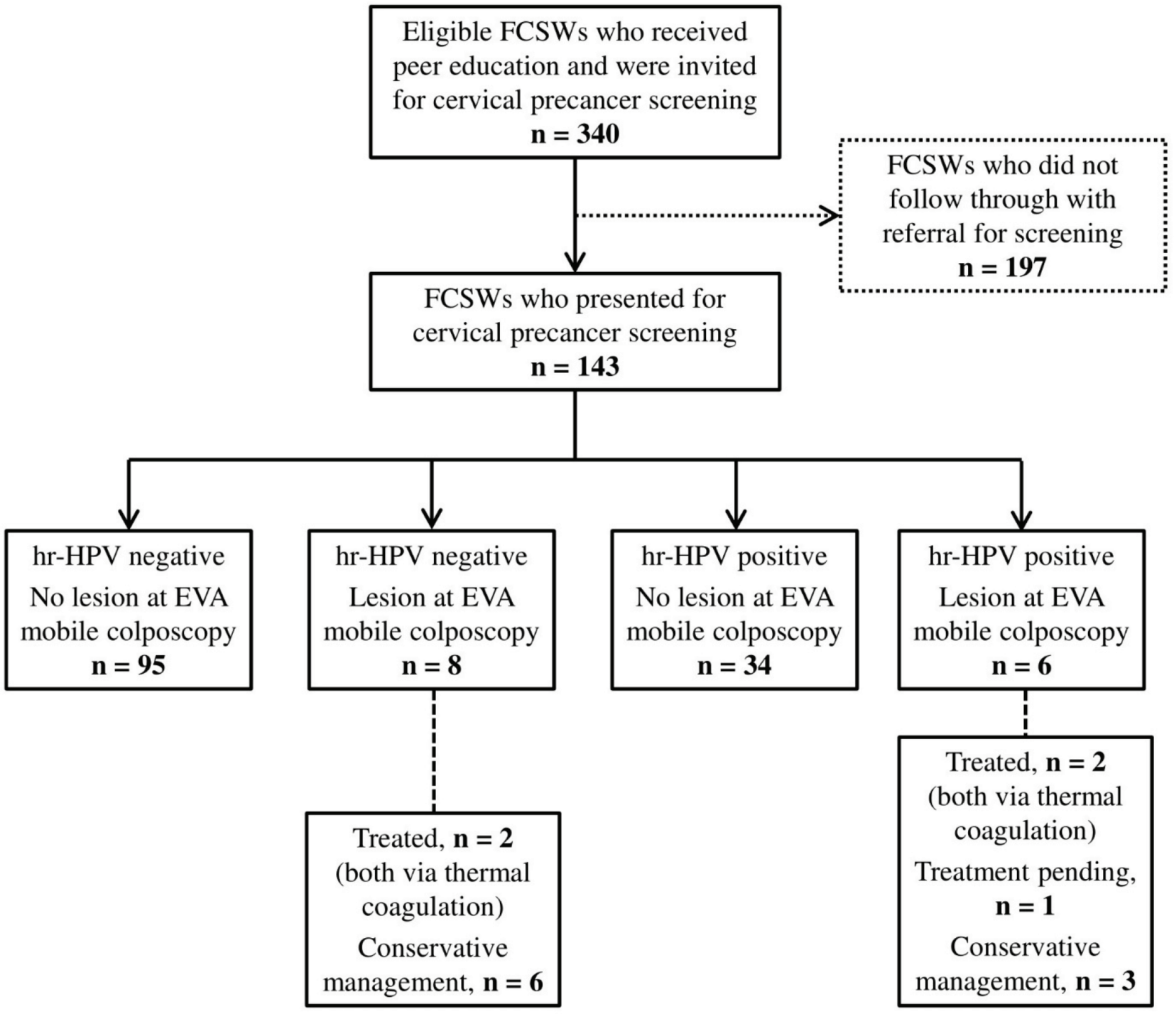
Flowchart for cervical precancer screening performed among FCSWs referred by peer educators using concurrent hr-HPV DNA testing and EVA mobile colposcopy. hr-HPV, high-risk human papillomavirus; EVA, enhanced visual assessment; FCSWs, female commercial sex workers.

Following sample collection, visual inspection was performed using the EVA system 3.0 (MobileODT, Tel Aviv, Israel) by the same screening nurse and in the same setting. This involved examining the cervix for abnormalities using a mobile colposcopy unit built around a smartphone that allows images to be taken and stored. Colposcopic images were captured before and after applying 5% acetic acid with a wait time of 90–120 seconds. The findings thereof were documented in accordance with the terminology of the International Federation of Cervical Pathology and Colposcopy (IFCPC), noting the adequacy of colposcopy, the type of TZ observed as well as the presence of clinically significant cervical lesion(s) [30, 31]. EVA positivity was noted for acetowhitening (thin/dense for minor/major changes), mosaicism (fine/coarse for minor/major changes) and punctuation (fine/coarse for minor/major changes). Nurse providers classified the TZ type seen at colposcopy using the 2011 criteria of the IFCPC, i.e., type 1 if the entire squamocolumnar junction (SCJ) could be seen (fully ectocervical), type 2 if the entire SCJ circumference could be visualised (partly or fully endocervical) or type 3 if the entire SCJ circumference could not be visualised (partly or fully endocervical). Women with cervical lesions on colposcopy who qualified for treatment by thermal coagulation and consented to it were treated by the trained nurses on site (Figure 1).

### Laboratory processing of cervical samples and hr-HPV DNA assays

Initial hr-HPV DNA testing was performed with the MA-6000 HPV assay system (Sansure Biotech Inc., Hunan, China), after which specimens obtained from screen-positive FCSWs were subjected to full genotyping using the AmpFire HPV DNA test platform (Atila BioSystems, Inc., Mountain View, USA). The samples were processed and run on the platforms strictly following the manufacturers’ instructions, details of which have been published elsewhere [32–35]. The MA-6000 assay system was run in its semiquantitative module and the genotypes identified were classified as follows: specific and separate identification of HPV16 and HPV18, and collective identification of HPV31/33/35/39/45/51/52/53/56/58/59/66/68 as *other* high-risk HPV types. The AmpFire HPV test platform was run in its full quantitative module and could thus distinguish among all the 15 aforementioned HPV genotypes separately.

### Triage, treatment and follow-up strategy

Due to resource limitations and to mitigate the risk associated with loss to follow-up, a screen-and-treat approach was used as much as possible [36]. Although hr-HPV testing was performed, the results could not be obtained in real time; thus, the results were used to retrospectively confirm the decision to treat and give follow-up recommendations. Women with cervical lesions could opt for onsite treatment even before the HPV results were available. Clinically significant cervical lesions were treated onsite using a handheld thermal coagulator (MTA-100 Thermal Ablation System, Liger Medical, United States). Eligibility for thermal coagulation was assessed strictly according to the WHO guidelines [37]. Briefly, a woman was deemed eligible for this treatment if there was no suspicion of invasive disease at colposcopy and an entirely visible lesion without extension into the endocervical canal, or a type 1 TZ lesion, or a type 2 TZ lesion for which the probe tip could achieve thorough ablation of the SCJ (reach the upper limit of the TZ) [31]. Women who showed minor changes (thin acetowhitening) on colposcopy were counselled about the option of conservative management (rescreening in 6 months to 1 year) as such lesions (probably CIN1) could regress spontaneously or undergo immediate thermal ablation, which could be overtreatment, but potentially lifesaving for those who might never get the opportunity to ever rescreen. Women who tested hr-HPV negative and showed no cervical abnormalities on EVA mobile colposcopy were advised to rescreen after 5 years (3 years for HIV-positive FCSWs). Participants who tested hr-HPV positive without corresponding cervical lesions were counselled to repeat HPV DNA testing in 1 year at a nearby facility or the CCPTC. Women who fell into these last two categories were counselled after HPV DNA tests were conducted at our central laboratory, not onsite.

### Statistical methods

The general sociodemographic and clinical details of the study participants are reported descriptively. Continuous variables with symmetric distributions such as age are described using means and standard deviations (SDs), while skewed discrete data such as parity are described using medians and interquartile ranges (IQRs). Categorical variables such as HIV status are described using frequencies and percentages. The prevalence of hr-HPV and clinically significant cervical lesions observed on EVA mobile colposcopy are presented as percentages with 95% confidence intervals (CIs). We employed univariable and multivariable binary logistic regression to explore factors associated with both hr-HPV infection and colposcopy ‘positivity’. Both multivariable models were fitted using the backward elimination procedure with an arbitrary probability threshold of 0.25 for variable removal. The effect sizes observed are expressed as crude and adjusted odds ratios (ORs and aORs, respectively) alongside their 95% CIs. All statistical analyses were performed using Stata version 18.5 (StataCorp LLC, College Station, TX, USA). Each null hypothesis was rejected at a two-tailed alpha level of 5%.

## Results

### Recruitment and triaging of FCSWs for cervical precancer screening

In total, 340 FCSWs received peer education concerning cervical cancer and were invited to undergo screening at designated community health centres (Figure 1). One hundred and ninety-seven of these FCSWs did not follow through with the referral for screening; thus, the final dataset analysed in this study comprised a total of 143 FCSWs, 52 screened in Aflao and 91 screened in Ho. This translated to an overall retention rate of 42.1% (95% CI, 36.8–47.3), broken down into 31.0% (52/168) among FCSWs invited in Aflao and 52.9% (91/172) among those invited in Ho.

### Sociodemographic and clinical characteristics of the FCSWs

The social, demographic and clinical characteristics of the study participants are summarised in Table 1. The FCSWs screened had a mean age of 31.9 (SD, 9.3) years and had given birth a median of 1 (IQR: 0, 2) time. A large majority (*n* = 116, 81.1%) had completed at least secondary education and were of the Christian faith (*n* = 132, 92.3%). Ninety-one FCSWs (63.6%) reported using any form of contraception at the time of screening, with about half (*n* = 71, 49.7%) using barrier contraception. Nine of the FCSWs (6.3%) self-reported being HIV positive or tested positive for HIV at screening. Fifty-nine (41.3%) had received some form of cervical cancer awareness prior to the index referral for screening, while 1 (0.7%) had undergone prior cervical precancer screening. None of the FCSWs had received HPV vaccination at the time of screening.

### Screening characteristics and outcomes

Table 2 summarises the gross outcomes observed at screening as well as prevalence estimates for outcomes recorded on EVA mobile colposcopy and hr-HPV DNA testing. A small minority of the FCSWs showed abnormal findings on gross inspection of the vulva (*n* = 4, 2.8%), vagina (*n* = 3, 2.1%) and cervix (*n* = 5, 3.5%). The commonest TZ type seen on EVA mobile colposcopy was type 3 (*n* = 101, 70.6%) followed by type 2 (*n* = 34, 23.8%).

**Figure 2.**
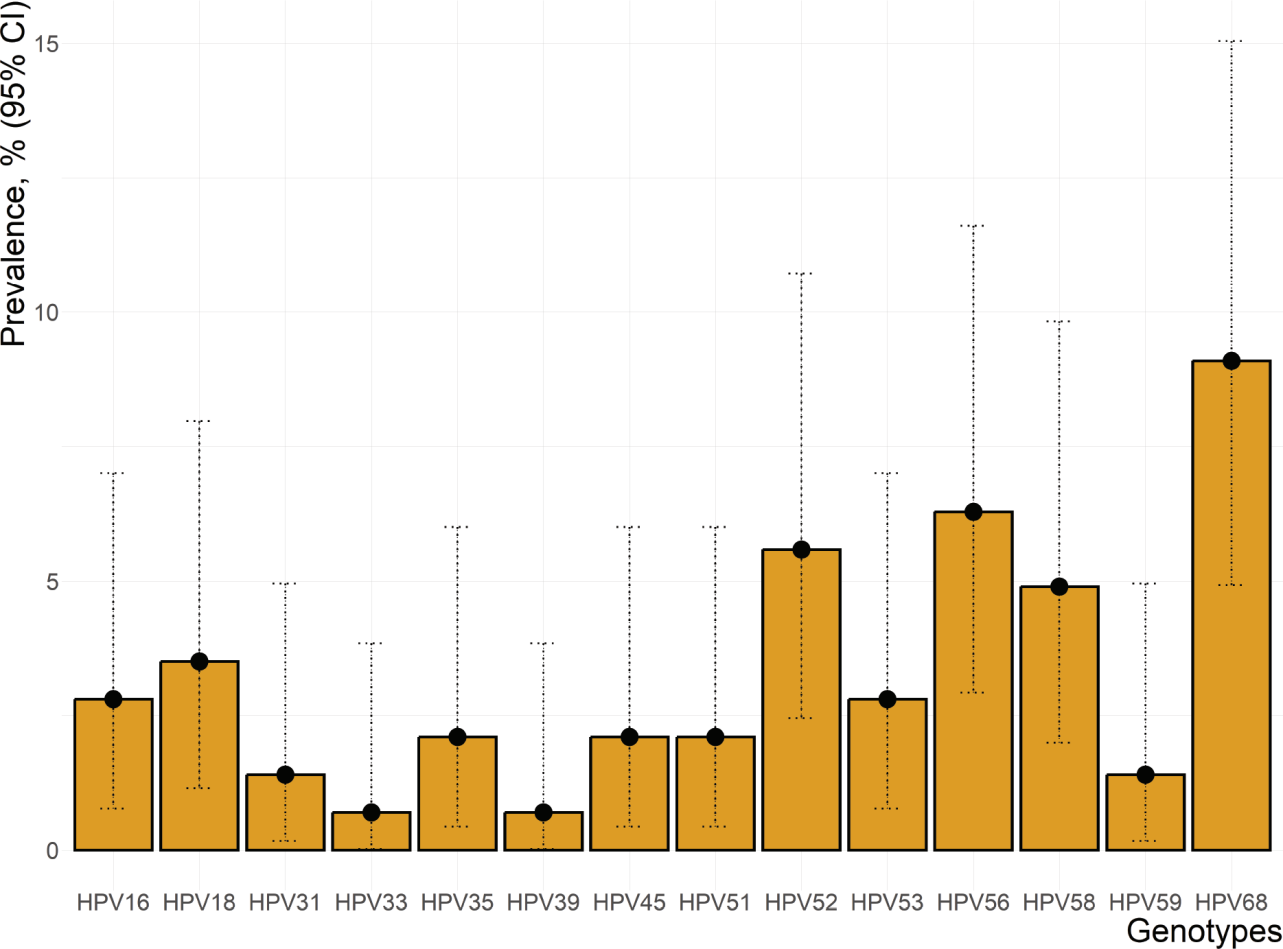
Prevalence of HPV infection disaggregated by individual genotypes among the FCSWs. Each error bar represents the 95% confidence interval for the prevalence estimate. Study participants could be infected with multiple HPV genotypes.

The prevalence estimate of cervical lesions on mobile colposcopy was 9.8% (95% CI, 4.9–14.7), while the prevalence of hr-HPV infection among the FCSWs was estimated as 28.0% (95% CI, 20.6–35.3). The prevalence of individual HPV genotypes (without considering FCSWs with single- versus mixed-genotype infections) is summarised in Figure 2. The commonest individual genotype was HPV68 (9.1%; 95% CI, 4.9–15.0), while the least common genotypes were HPV33 and HPV39 (each 0.7%; 95% CI, 0.0–3.8). The commonest genotypes recorded among FCSWs with single hr-HPV types were HPV16 and HPV52 (2.1%; 95% CI, 0.4–6.0) each, followed by HPV56 and HPV68 (1.4%; 95% CI, 0.2–5.0). Similarly, the commonest genotypes recorded among FCSWs with mixed hr-HPV infection were HPV68 + HPV52 and HPV58 + HPV56, with prevalence estimates of 2.1% (95% CI, 0.4–6.0) each. Triple-genotype hr-HPV infections were recorded among 0.7% (95% CI, 0.0–3.8) of the FCSWs, each for HPV68 + HPV52 + HPV53 and HPV68 + HPV45 + HPV53 (Table 2).

### Exploring factors associated with hr-HPV infection and cervical lesions on EVA mobile colposcopy among the FCSWs

Table 3 summarises the results of binary logistic regression analyses of variables associated with hr-HPV infection and clinically significant lesions on EVA mobile colposcopy. In the univariable analysis, FCSWs screened in Aflao showed threefold higher odds of hr-HPV infection than those screened in Ho (OR, 2.97; 95% CI, 1.40–6.32). The adjusted analysis involved a model that controlled for age, religious affiliation, education level, marital status, HIV status, oral-genital contact, past gynaecological surgery, past contraceptive use, cervical cancer awareness, current use of oestrogen/nonoestrogen-containing contraceptives and TZ type. FCSWs screened in Aflao maintained the threefold higher odds of hr-HPV infection observed in the univariable model (aOR, 3.33; 95% CI, 1.18–9.40; *p*-value = 0.023) and FCSWs with cervices that showed clinically significant lesions on EVA mobile colposcopy were approximately five times more likely to test hr-HPV positive (aOR, 4.64; 95% CI, 1.09–19.83; *p*-value = 0.038) compared to those whose cervices were EVA colposcopy ‘negative’. Condom use at the time of screening was independently associated with decreased odds of hr-HPV infection (aOR, 0.14; 95% CI, 0.04–0.49; *p*-value = 0.002).

On their own, none of the variables assessed showed any significant association with clinically significant lesions on EVA mobile colposcopy. However, in the adjusted analysis, HIV-positive FCSWs (aOR, 9.95; 95% CI, 1.19–83.37; *p*-value = 0.034) and FCSWs who engaged in oral-genital contact (aOR, 5.56; 95% CI, 1.35–22.94; *p*-value = 0.018) showed higher odds of cervical lesions on EVA mobile colposcopy after controlling for age, parity, cervical cancer awareness and manual-genital contact (Table 3).

## Discussion

A worrying finding of this study was that while FCSWs peer educated and referred from Aflao were significantly less likely than those from Ho to follow through and actually be screened (chi-squared *p*-value <0.001), they showed threefold higher odds of hr-HPV infection compared to the latter cohort (aOR, 3.33; 95% CI, 1.18–9.40). The overall retention rate of 42.1% indicates that nearly half of the FCSWs who received peer education and were referred were eventually screened. This is still below the target set by the WHO of 70% eligible women to be screened for cervical precancer, and compared to rates seen in opportunistic screening strategies, this is a significant finding, given the high risk of STIs (including HPV) among FCSWs. In sub-Saharan Africa, the estimated coverage of cervical screening services has been consistently low, ranging from below 1% to 5.3% [38, 39]. Again, the fact that more than half of the FCSWs who received peer counselling did not get screened points to the need for additional strategies to improve screening uptake. For instance, providing screening services at locations and times that are convenient for FCSWs, reducing the cost of screening and addressing stigma and discrimination in healthcare settings could further increase screening uptake. Recent reports from the continent highlight the feasibility of integrating text messaging and self-sampling strategies into potential testing strategies for FCSWs [25].

The crude overall HPV prevalence identified among the FCSWs was approximately twice that reported among a group of women screened in an outpatient setting in Accra [40]. The HPV prevalence among our study participants again exceeded the expected prevalence reported by the WHO [41] for unscreened women in West Africa and was not too dissimilar from that recorded among women screened elsewhere in the Volta region [42]. However, our prevalence estimate was approximately a third of that reported among FCSWs in Senegal (79.8%) [43]. This vast difference in prevalence could be due to the fact that the Senegalese study included many FCSWs as young as 15 and 18 years, while women in our cohort were relatively older. It is well known that HPV infections are more likely to be persistent among older women. This study also highlighted variations in individual HPV genotypes, with the most common genotypes being HPV68 and HPV56, compared to community-dwelling women in the Volta region among whom researchers [42] most commonly found HPV16 and HPV52 (Figure 2). The specific genotype distribution also differed from that presented in a systematic review and meta-analysis of HPV prevalence among FCSWs in the sub-Saharan region, which found the most common types to be HPV16, HPV52 and HPV53 [16]. Due to financial limitations, we could only perform full genotyping using the AmpFire platform for FCSWs who tested positive for the combined ‘*other*’ hr-HPV classification on MA-6000 to discern among those. All but one (HPV66) of the 15 hr-HPV genotypes detectable using MA-6000/AmpFire were represented in our study cohort. This finding of a difference in genotypic distribution from that more prevalent in the general population, combined with the zero rate of HPV vaccination among the study participants, underscores a significant implementation gap in cervical cancer prevention among FCSWs and could highlight a need to take another look at the use of vaccines such as Cervarix and Gardasil-4 when targeting high-risk groups like FCSWs in Ghana in the near future.

Our study again underscores the significant associations between ongoing condom use (aOR, 0.14; 95% CI, 0.04–0.49) and hr-HPV infections among FCSWs in the Volta Region of Ghana, highlighting a need to promote continuous condom use among FCSWs during interactions with patrons. These results align well with what is known in the literature between these two variables, with a Danish cohort of FCSWs who reported ongoing condom at testing showing a significantly decreased risk of HPV infection (OR, 0.2; 95% CI, 0.1–0.6) [44]. Furthermore, a subgroup meta-analysis also showed an overall higher pooled hr-HPV prevalence among studies in which ≤50% FCSWs reported frequent and habitual condom use compared with when a rate of >50% was reported [45]. We also found a significant association between HIV positivity and clinically significant lesions at colposcopy. While HIV testing could not be performed for all FCSWs at screening due to financial and logistical constraints (and we had a mix of self-reported and clinically confirmed cases), this association is well-reported in the literature [3]. This could be due to the immunosuppressive effects of HIV, which can impair the body’s ability to clear HPV infections, implying that FCSWs living with HIV may require more frequent cervical precancer screening and follow-up.

The finding of 9.8% clinically significant lesion on EVA mobile colposcopy was very similar to the prevalence found in prior studies conducted by our research team on women in the general population [36, 46]. This was contrary to the expectation of a higher prevalence of EVA ‘positive’ lesions given the high HPV prevalence among FCSWs. On the other hand, given that HPV16 and HPV18 are the most virulent genotypes [3] and result in more than 70% of pre-invasive lesions globally, our finding of a relatively low prevalence for these types in our cohort could potentially be protective against EVA ‘positive’ lesions in our study sample. In another study of VIA positivity among FCSWs in Uganda [47], the ‘naked-eye’ VIA ‘positivity’ rate was as low as 6%. Apart from the added benefit of using EVA mobile colposcopy for visual inspection in our study, the nurses who performed visual inspection in our study were arguably more experienced. It also remains to be investigated whether this relatively low prevalence of clinically significant lesions among the FCSWs would similarly reflect in a low prevalence of histopathologically confirmed precancerous lesions.

As shown in the present study, peer educators and NGOs are invaluable in identifying and responding to the screening needs of FCSWs; however, there is a paucity of data from program implementation, which limits their evaluation and utilization. Their operations also rely on external funding, hindering program sustainability and with potential consequences for FCSW health. Despite the benefits derived from using peer educators as a bridge between FCSWs and screening, follow-up was a major challenge as has been documented in numerous programs. [48–50] Apart from the opportunistic and nonorganised nature of cervical precancer screening in Ghana, the itinerant nature of FCSWs [48] was another obvious barrier. A protocol of concurrent screening and a screen-and-treat approach was applied to overcome this barrier; however, following post-treatment recommendations and following up with those recommended to undergo conservative management would be challenging, outside a program (the mTTWI) context [51]. The see-and-treat approach appears to be more appropriate for high-risk and vulnerable populations like FCSWs because getting them for triage and follow-up can be quite challenging. Evidently, once an organised protocol is set up, there is a need to develop a robust follow-up system tailored to high-risk populations like FCSWs to ensure program effectiveness and sustainability, taking into consideration financial and human resource limitations. While for logistical reasons, peer educator engagement in such a capacity is easier done with NGOs like Pro-Link, another long-term strategy would be to integrate FCSW-specific service into public health facilities, alongside health navigators, such that FCSWs can express their needs in a nonstigmatizing and non-judgemental environment. This would, however, start with destigmatizing and decriminalizing their activities. It is also quite plausible that strengthening relationships between FCSWs and healthcare providers, promoting social norms around screening and using self-sampling screening protocols could facilitate screening utilization among FCSWs [16].

## Strengths and limitations

The present study had a number of strengths. First, it is one of the first studies to investigate the prevalence of hr-HPV infection and cervical lesions among FCSWs in Ghana and to model factors associated with each of these screening outcomes. We collaborated with Pro-Link, an NGO that has worked with FCSWs in Ghana for several years, and thus built the trust needed to ensure that the data presented here were accurate. The sociodemographic data were collected by CCPTC staff with the assistance of Pro-Link who were well acquainted with the FCSWs and their way of life. Also, unlike other studies conducted in the sub-Saharan region that included FCSWs as young as 15–18 years of age, more than 80% of FCSWs in our study sample were ≥25 years of age, and thus represented the age range more likely to show persistent rather than transient hr-HPV infection. Again, we used a concurrent approach to screen, followed by treatment: while many prior studies conducted in the sub-Saharan region neither reported using this approach nor performed treatment for screen positives. Our study, however, has a few limitations. First, due to our low study sample size and the low number of EVA positivity cases, our ability to assess factors associated with EVA positivity is limited. This could be a reason why no significant factors were found to be associated with adverse EVA findings. In addition, the criterion for adjudging positivity on VIA or mobile colposcopy is commonly the presence of acetowhitening. Acetowhitening may be due to immature metaplasia, inflammation, subclinical papillomavirus infection or cervical intraepithelial neoplasia (CIN). Biopsies for histopathology would have been useful to confirm precancerous (CIN2+) lesions. Furthermore, we screened any woman who self-identified as FCSW after referral by a peer educator, without formal verification. While the risk of study population contamination was low, a few non-FCSWs could have self-identified as FCSWs to benefit from screening, potentially biasing our results toward the null.

## Conclusion

Our study underscores the urgent need for comprehensive and targeted interventions to reduce the burden of hr-HPV and the potential for cervical precancer/cancer among FCSWs in Ghana. Such interventions should include improving their knowledge and uptake of cervical precancer screening, continuous peer education about the risks of hr-HPV and cervical cancer and improving their access to HPV vaccination. We further highlight the importance of promoting continuous condom use among FCSWs during interactions with patrons and considering HIV status in the development and implementation of cervical precancer screening programs for FCSWs. The finding of a different genotypic distribution from that more prevalent in the general population, combined with the zero rate of HPV vaccination could highlight a need to take another look at the use of routine HPV vaccines when targeting high-risk groups like FCSWs in Ghana in the near future. Further studies are needed to better understand the observed associations and to develop effective and sustainable strategies for HPV prevention and control among FCSWs in Ghana.

## Conflicts of interest

The authors declare that they have no competing interests to declare.

## Ethical approval and consent to participate

The study complied with the Declaration of Helsinki (1964) and its later amendments. Informed consent was sought from FCSWs prior to questionnaire administration, sample collection and cervical screening. Ethical clearance was given by the Ethical Review Committee of the Catholic Hospital, Battor (approval no. CHB-ERC 0120/06/22). The study was conducted in an environment with no form of coercion and volunteers were adequately informed of the purpose, nature and procedures of the study procedures.

## Availability of data and materials

The dataset supporting the conclusions of this article is included within the article and its additional files.

## Author contributions

Conceptualization and study design: KE, ET, CMW, VK and JEA; Screening and data collection: ET, CMW, EACM, VK, SK, SD and KE; Data management and formal analysis: JEA, NOME, SD, ET, CMW, SK and KE; Writing – original draft: LAM, JEA, ETD, NOME, ET, PKA and KE. All the authors read and approved the manuscript in its current form.

## Figures and Tables

**Table 1.** Sociodemographic and clinical characteristics (stratified by screening site) of 143 FCSWs screened using concurrent hr-HPV DNA testing and EVA mobile colposcopy.

Sociodemographic and clinical details	Screening site		Overall (*n* = 143)
	Ho (*n* = 91)	Aflao (*n* = 52)	
Age, mean (SD)	32.6 (9.4)	30.6 (9.2)	31.9 (9.3)
Age group, *n* (%)			
<25	16 (17.6)	19 (36.5)	35 (24.5)
25–34	42 (46.2)	15 (28.9)	57 (39.9)
35–44	21 (23.1)	14 (26.9)	35 (24.5)
45–54	9 (9.9)	3 (5.8)	12 (8.4)
≥55	3 (3.3)	1 (1.9)	4 (2.8)
Age at menarche,^1^ mean (SD)	-	15.9 (2.2)	15.9 (2.2)
Marital status,* n* (%)			
Single	39 (42.9)	27 (51.9)	66 (46.2)
Has a steady partner	31 (34.1)	20 (38.5)	51 (35.7)
Married/cohabiting	14 (15.4)	1 (1.9)	15 (10.5)
Divorced	6 (6.6)	2 (3.9)	8 (5.6)
Widowed	1 (1.1)	2 (3.9)	3 (2.1)
Number of lifetime pregnancies, median (IQR)	1 (0, 2)	1 (0, 2)	1 (0, 2)
Number of births, median (IQR)	0 (0, 2)	1 (0, 2)	1 (0, 2)
Highest education level, *n* (%)			
No formal education	5 (5.5)	5 (9.6)	10 (7.0)
Elementary education	6 (6.6)	11 (21.2)	17 (11.9)
Secondary education	60 (65.9)	33 (63.5)	93 (65.0)
Tertiary education	19 (20.9)	2 (3.9)	21 (14.7)
Vocational/technical/other	1 (1.1)	1 (1.9)	2 (1.4)
Religious faith, *n* (%)			
Christian	88 (96.7)	44 (84.6)	132 (92.3)
Islam	3 (3.3)	1 (1.9)	4 (2.8)
Traditional/other	0 (0.0)	7 (13.5)	7 (4.9)
Current contraceptive use*, *n* (%)	55 (60.4)	36 (69.2)	91 (63.6)
Withdrawal/rhythm	7 (7.7)	0 (0.0)	7 (4.9)
Condom	42 (46.2)	29 (55.8)	71 (49.7)
Combined pill	6 (6.6)	1 (1.9)	7 (4.9)
Progesterone only pill	1 (1.1)	5 (9.6)	6 (4.2)
Depot Provera	6 (6.6)	7 (13.5)	13 (9.1)
Implant	9 (9.9)	2 (3.9)	11 (7.7)
Past contraceptive use*, *n* (%)	55 (60.4)	40 (76.9)	95 (66.4)
Withdrawal/rhythm	9 (9.9)	0 (0.0)	9 (6.3)
Condom	31 (34.1)	26 (50.0)	57 (39.9)
Combined pill	10 (11.0)	4 (7.7)	14 (9.8)
Progesterone only pill	3 (3.3)	6 (11.5)	9 (6.3)
Depot Provera	9 (9.9)	12 (23.1)	21 (14.7)
Implant	13 (14.3)	3 (5.8)	16 (11.2)
NHIS coverage, *n* (%)	67 (73.6)	46 (88.5)	113 (79.0)
Ever smoked, *n* (%)	5 (5.5)	6 (11.5)	11 (7.7)
HIV status, *n* (%)			
Positive	3 (3.3)	6 (11.5)	9 (6.3)
Negative	73 (80.2)	19 (36.5)	92 (64.3)
Unknown	15 (16.5)	27 (51.9)	42 (29.4)
Existing medical condition* (yes), *n* (%)	20 (22.0)	13 (25.0)	33 (23.1)
Hypertension, *n* (%)	5 (5.5)	6 (11.5)	11 (7.7)
Asthma, *n* (%)	5 (5.5)	0 (0.0)	5 (3.5)
Diabetes mellitus, n (%)	2 (2.2)	1 (1.9)	3 (2.1)
Prior gynaecological surgery, *n* (%)	14 (15.4)	4 (7.7)	18 (12.6)
Duration as a sex worker, years; median (IQR)	-	4 (2, 7)	4 (2, 7)
No. of clients/patrons per week, median (IQR)	-	5 (3, 10)	5 (3, 10)
Vaginal herbal applications (yes), *n* (%)	56 (61.5)	15 (28.9)	71 (49.7)
Type(s) of sexual activity*, *n* (%)			
Genital-to-genital contact	61 (67.0)	47 (90.4)	108 (75.5)
Oral-genital contact	27 (29.7)	13 (25.0)	40 (28.0)
Manual-genital contact	22 (24.2)	3 (5.8)	25 (17.5)
Cervical cancer awareness, *n* (%)	37 (40.7)	22 (42.3)	59 (41.3)
Prior cervical precancer screening, *n* (%)	0 (0.0)	1 (1.9)	1 (0.7)

HPV, human papillomavirus; HIV, human immunodeficiency virus; SD, standard deviation; IQR, interquartile range; EVA, enhanced visual assessment

1 Reported only for 49 FCSWs, all screened at Aflao

* Multiple-choice item

**Table 2.** Screening characteristics and outcomes of 143 FCSWs screened using concurrent hr-HPV DNA testing and EVA mobile colposcopy.

Gross screening characteristic	Estimate
Abnormal vulval inspection findings, n (%)	4 (2.8)
Abnormal vaginal inspection findings, *n* (%)	3 (2.1)
Abnormal cervical inspection finding, *n* (%)	5 (3.5)
	
TZ type^α^ on EVA mobile colposcopy	
1	8 (5.6)
2	34 (23.8)
3	101 (70.6)
Screening outcome (prevalence estimates)	
EVA mobile colposcopy ‘positive’, % (95% CI)	9.8 (4.9–14.7)
hr-HPV positive, % (95% CI)	28.0 (20.6–35.3)
Single versus mixed hr-HPV infections, % (95% CI)	
HPV16 only	2.1 (0.4–6.0)
Other hr-HPV genotypes^β^	25.2 (18.3–33.1)
HPV52 only	2.1 (0.4–6.0)
HPV53 only	0.7 (0.0–3.8)
HPV56 only	1.4 (0.2–5.0)
HPV68 only	1.4 (0.2–5.0)
HPV18 + HPV52	0.7 (0.0–3.8)
HPV35 + HPV45	0.7 (0.0–3.8)
HPV35 + HPV68	1.4 (0.2–5.0)
HPV35 + HPV59	0.7 (0.0–3.8)
HPV45 + HPV68	0.7 (0.0–3.8)
HPV68 + HPV52	2.1 (0.4–6.0)
HPV68 + HPV53	0.7 (0.0–3.8)
HPV58 + HPV56	2.1 (0.4–6.0)
HPV68 + HPV52 + HPV53	0.7 (0.0–3.8)
HPV68 + HPV45 + HPV53	0.7 (0.0–3.8)

hr-HPV, high-risk human papillomavirus; TZ, transformation zone; CI, confidence interval; EVA, enhanced visual assessment

α Transformation zone types

TZ1: The entire circumference of the squamocolumnar junction is visible; fully ectocervical

TZ2: The entire circumference of the squamocolumnar junction is visible; partly or fully endocervical

TZ3: The entire circumference of the squamocolumnar junction is not visible; partly or fully endocervical

β Full genotyping (using the AmpFire platform) was completed for 26 of these 36 FCSWs identified with *other* hr-HPV genotypes on MA-6000 testing

**Table 3.** Exploratory logistic regression analyses of factors associated with hr-HPV infection and colposcopy ‘positivity’ among the FCSWs.

Variable	hr-HPV infection				Colposcopy ‘positivity’			
	Univariable models		Adjusted model		Univariable models		Adjusted model	
	OR (95% CI)	*p*-value	aOR (95% CI)	*p*-value	OR (95% CI)	*p*-value	aOR (95% CI)	*p*-value
Age, years (continuous)	0.99 (0.95–1.03)	0.641	-	-	0.93 (0.86–1.01)	0.080	0.93 (0.86–1.01)	0.083
Age group, years (categorical)								
<30	1.49 (0.71–3.11)	0.288	2.21 (0.72–6.84)	0.167	1.89 (0.60–5.93)	0.278	-	-
≥30 (ref.)	1.00	-	1.00		1.00	-	-	-
Parity (discrete)	0.90 (0.70–1.16)	0.406	-	-	0.70 (0.41–1.22)	0.213	0.67 (0.39–1.16)	0.151
Parity (categorical)								
0 (ref.)	1.00	-	-	-	1.00	-	-	-
≥1	1.07 (0.51–2.22)	0.863	-	-	0.61 (0.20–1.87)	0.389	-	-
Religious faith								
Christian	0.29 (0.08–1.01)	0.052	0.20 (0.03–1.17)	0.073	-	-	-	-
Traditional/Islam/other (ref.)	1.00	-	1.00	-	-	-	-	-
Education level								
Below secondary	1.79 (0.76–4.23)	0.184	3.42 (0.92–12.78)	0.067	0.28 (0.03–2.21)	0.226	-	-
Secondary school or higher (ref.)	1.00	-	1.00	-	1.00	-	-	-
NHIS coverage (yes versus no)	2.24 (0.79–6.35)	0.128	-	-	0.63 (0.18–2.17)	0.466	-	-
Marital status								
Married/has a steady partner (ref.)	1.00	-	1.00	-	1.00	-	-	-
Single/divorced/widowed	1.63 (0.77–3.45)	0.198	2.12 (0.78–5.74)	0.139	1.61 (0.51–5.08)	0.413	-	-
HIV status (positive versus negative/unknown)	3.54 (0.90–13.92)	0.071	4.67 (0.44–49.01)	0.199	2.90 (0.54–15.58)	0.213	9.95 (1.19–83.37)	0.034*
Screening site (Aflao versus Ho)	2.97 (1.40–6.32)	0.005*	3.33 (1.18–9.40)	0.023*	1.35 (0.44–4.14)	0.596	-	-
Vaginal herbal applications (yes versus no)	0.67 (0.32–1.40)	0.288	-	-	1.02 (0.34–3.06)	0.978	-	-
Type(s) of sexual activity^α^								
Genital-to-genital contact	1.76 (0.70–4.43)	0.230	-	-	2.06 (0.44–9.70)	0.359	-	-
Oral-genital contact	1.59 (0.73–3.50)	0.245	2.61 (0.76–9.01)	0.129	2.10 (0.68–6.48)	0.199	5.56 (1.35–22.94)	0.018*
Manual-genital contact	0.59 (0.21–1.71)	0.332	-	-	0.34 (0.04–2.70)	0.305	0.21 (0.02–1.90)	0.164
Past gynaecological surgery	1.34 (0.47–3.85)	0.589	4.29 (0.99–18.67)	0.052	0.51 (0.06–4.13)	0.525	-	-
Current contraceptive use	0.70 (0.33–1.47)	0.343	-	-	2.25 (0.60–8.45)	0.231	-	-
Condom	0.50 (0.24–1.06)	0.072	0.14 (0.04–0.49)	0.002*	-	-	-	-
Combined (oestrogen-containing) pill	1.03 (0.30–3.51)	0.958	4.70 (0.70–31.56)	0.111	-	-	-	-
Other (nonoestrogen-containing)	0.91 (0.41–2.01)	0.814	2.11 (0.67–6.66)	0.202	-	-	-	-
Past contraceptive use	0.58 (0.27–1.24)	0.161	0.36 (0.12–1.06)	0.064	1.29 (0.38–4.36)	0.678	-	-
Condom	0.50 (0.24–1.06)	0.072	-	-	-	-	-	-
Combined (oestrogen-containing) pill	2.01 (0.43–9.40)	0.377	-	-	-	-	-	-
Other (nonoestrogen-containing)	2.17 (0.92–5.09)	0.075	-	-	-	-	-	-
Cervical cancer awareness (yes versus no)	1.24 (0.59–2.59)	0.571	2.62 (0.98–7.00)	0.056	0.36 (0.09–1.34)	0.126	0.28 (0.06–1.22)	0.090
TZ type seen at colposcopy								
Type 1	1.07 (0.20–5.65)	0.937	-	-	3.88 (0.67–22.43)	0.130	-	-
Type 2	2.25 (0.99–5.11)	0.054	2.72 (0.89–8.25)	0.078	1.55 (0.44–5.51)	0.498	-	-
Type 3 (ref.)	1.00	-	1.00	-	1.00	-	-	-
EVA mobile colposcopy ‘positive’	2.10 (0.68–6.48)	0.199	4.64 (1.09–19.83)	0.038*	-	-	-	-

TZ, transformation zone; CI, confidence interval; OR, odds ratio; hr-HPV, high-risk human papillomavirus; ref., reference category

* Statistically significant

α Multiple-choice item
